# Modulation of the glutamatergic transmission by Dopamine: a focus on Parkinson, Huntington and Addiction diseases

**DOI:** 10.3389/fncel.2015.00025

**Published:** 2015-03-02

**Authors:** Fabrizio Gardoni, Camilla Bellone

**Affiliations:** ^1^Department of Pharmacological and Biomolecular Sciences, University of MilanoMilano, Italy; ^2^Department of Fundamental Neuroscience, University of LausanneLausanne, Switzerland

**Keywords:** Dopamine, NMDA receptor, AMPA receptors, Addiction, Parkinson disease, Huntington disease

## Abstract

Dopamine (DA) plays a major role in motor and cognitive functions as well as in reward processing by regulating glutamatergic inputs. In particular in the striatum the release of DA rapidly influences synaptic transmission modulating both AMPA and NMDA receptors. Several neurodegenerative and neuropsychiatric disorders, including Parkinson, Huntington and addiction-related diseases, manifest a dysregulation of glutamate and DA signaling. Here, we will focus our attention on the mechanisms underlying the modulation of the glutamatergic transmission by DA in striatal circuits.

## Introduction

Dopamine (DA) is a catecholamine that acts as neuromodulator by playing an important role in motor and cognitive functions as well as in reward processing.

Our major understanding of the DA transmission derives from studies of the midbrain DA system that comprehend both Substantia Nigra pars compacta (SNc-A9) and Ventral Tegmental Area (VTA—A10). The former is at the origin of the nigrostriatal pathway where DA neurons project to the dorsal striatum and play a central role in controlling fine motor functions. Instead DA neurons within the VTA form the mesostriatal pathway and project to the ventral striatum (or Nucleus accumbens, NaC) exerting an important role in reward processing (Paillé et al., 2010; Tritsch and Sabatini, 2012). How does DA shape all these different functions in the brain? In both circuitries, DA acts as a neuromodulator regulating the glutamatergic inputs onto the principal neurons and therefore controlling the striatal output. More than 95% of striatal neurons are represented by Medium Spiny Neurons (MSNs; Kreitzer, 2009) that form asymmetric synapses with glutamatergic projections and symmetric contacts at the DA inputs. Therefore, the activity of DA neurons and the consequent release of DA in the proximity of the synaptic cleft rapidly influences synaptic transmission, intrinsic excitability and dendritic integration (Tritsch and Sabatini, 2012), partially explaining the different functions of DA in the brain. Importantly DA can modulate glutamatergic transmission by the convergence effect onto MSNs, by acting on D2-R located presynaptcally on Glutamatergic inputs or by modulating excitatory inputs onto GABAergic and Cholinergic interneurons.

**Figure 1:**
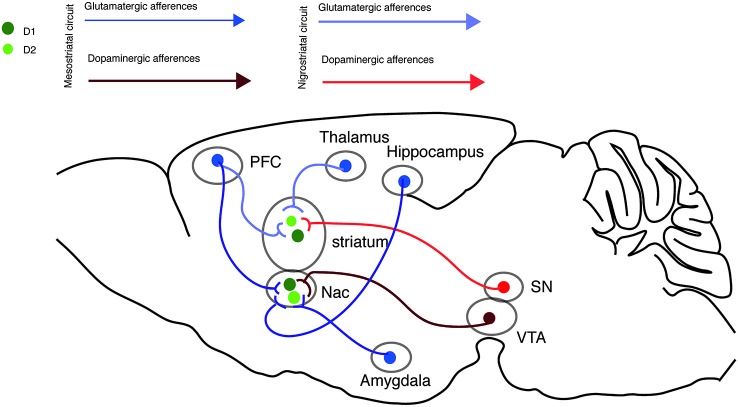
**Nigrostriatal and Mesostriatal circuits**. Sagittal view of the excitatory inputs onto the nigrostriatal and mesostriatal circuits.

Interestingly, several neurodegenerative and neuropsychiatric disorders, including Parkinson, Huntington and addiction-related diseases, manifest a dysregulation of glutamate and DA signaling within the striatum. In this review, we will focus our attention on the mechanisms underlying the modulation of the glutamatergic transmission by DA in the nigrostriatal and mesostriatal circuitries (Figure 1).

## Nigrostriatal circuit

DA neurons of the SNc project to the dorsal striatum. This structure is mainly populated by MSNs that are classified in two populations according to their axonal projections and DA receptor expression. DA receptor type 1 (D1R)-containing MSNs form the direct pathway and send their axons to the GABAergic output nuclei of the basal ganglia, the internal segment of the Globus Pallidus (GPi) and the Substantia Nigra pars reticulata (SNr), which in turn send their afferences to the motor nuclei of the thalamus. DA receptor type 2 (D2R)-containing MSNs constitute the indirect pathway and send their axons to the external segment of the Globus Pallidus (GPe), which in turn project to the glutamatergic neurons of the Sub-Thalamic Nucleus (STN). STN neurons then send their axons to the basal ganglia output nuclei (GPi and SNr) where they form excitatory synapses on the inhibitory output neurons. Activation of the direct and indirect pathway exerts an opposite effect on movement: activation of the direct pathway disinhibits the thalamocortical projections and leads to activation of the cortical premotor circuits facilitating movements. The activation of the indirect pathway instead inhibits the thalamocortical projection neurons reducing the premotor drive and inhibiting movements (Kreitzer and Malenka, 2008). Interestingly this model has been recently challenged and it has been proposed that the two pathways are structurally and functionally intertwined (Dunah and Standaert, 2001; Calabresi et al., 2014).

By acting on D1R or D2R, DA differently modulates the activity of the direct and indirect pathway both controlling the excitability of MSNs in the striatum and governing synaptic plasticity at different glutamatergic inputs. The majority of glutamatergic afferents onto the dorsal striatum originates in the cortex and thalamus. While corticostriatal afferences may carry motor and cognitive information, thalamostriatal ones convey information for the reward saliency and the wakefulness (Huerta-Ocampo et al., 2014). Despite this view, both corticostriatal and thalamostriatal terminals form synaptic contacts with D1 and D2 MSNs and the convergence of their inputs suggests that they are similarly involved in activation of the MSNs.

Profound functional differences in these pathways have been found, suggesting input-dependent differences in synaptic functions (Smeal et al., 2008). Future studies are needed to investigate the input segregation onto the direct and indirect striatal pathways and their functional implications.

## Mesostriatal circuit

This circuit originates in the VTA where DA neurons project to D1 and D2 MSNs of the ventral striatum. Although the presence of D1 and D2 MSNs in the ventral striatum is well established, there are several evidences showing that projections from the NAc may not be so segregated as for the dorsal striatum. Indeed, it has been shown that both D1 and D2 MSNs project to the ventral pallidum, while D1 MSNs can also directly project to the VTA (Lu et al., 1998; Zhou et al., 2003; Smith et al., 2013). Despite these differences, it is well established that D1 and D2 MSNs in the NAc exhibit different electrophysiological properties (Paillé et al., 2010; Pascoli et al., 2011b, 2014b) and respond differently to VTA stimulation (Grueter et al., 2010; Paillé et al., 2010). Despite this clear segregation of D1 and D2 containing MSN, it should be mention the existence of a small population of neurons containing both D1Rs and D2Rs (Matamales et al., 2009).

Similarly to the nigrostriatal circuit, DA modulates and integrates glutamatergic synaptic inputs from the prefrontal cortex, the amygdala and the hippocampus. Interestingly, different forms of synaptic plasticity have been described at different excitatory inputs onto D1 and D2 MSNs suggesting that specific pattern of neuronal activity coinciding with DA signal are needed for specific reward-related behavioral outcomes (Paillé et al., 2010; Pascoli et al., 2014b).

## DA receptors and signaling pathways

DA transmission is mediated by Guanine nucleotide binding Protein Coupled Receptors (GPCRs). They are metabotropic receptors with seven transmembrane domains coupled to G-proteins that lead to the formation of second messengers and the activation or inhibition of subsequent signaling cascades. Although five different DA receptors have been cloned so far, it is possible to classify them in two major populations according to their structures and their pharmacological properties: (a) D1-like receptors (D1 and D5) which stimulate cAMP production; and (b) D2-like receptors (D2, D3 and D4) which reduce the intracellular cAMP levels. The ability of D1-like and D2-like receptors to modulate in opposite directions the concentration of cAMP, and thus the downstream signal transduction, depends on their interaction with specific G proteins.

D1-like receptors are the most highly expressed DA receptor in the brain, are mainly localized within the forebrain and, compared to the D2-like family, have a highly conserved sequence (Tritsch and Sabatini, 2012). Binding of DA with D1-like receptors leads to an increase in the adenylyl cyclase activity and a consequent rise in cAMP levels. This pathway induces the activation of protein kinase A (PKA) and the phosphorylation of different substrates as well as the induction of immediate early gene expression that contribute to the overall D1R response (Beaulieu and Gainetdinov, 2011). DARPP-32 (DA and cAMP-regulated phosphoprotein, 32kDa) is one of the most studied PKA substrates activated by DA and provides a mechanism for integrating information at dopaminoceptive neurons (Svenningsson et al., 2004). Via the control of Protein Phosphatase-1 (PP-1), DARPP-32 regulates neuronal excitability as well as glutamatergic transmission. Activation of the cAMP/PKA/DARPP-32 pathway indeed increases the opening of the L-type Ca2+ channels promoting the transition of MSNs to a higher level of excitability (Vergara et al., 2003). At the same time, the activation of this pathway promotes the phosphorylation of both AMPARs and NMDARs providing a mechanism for the direct control of glutamatergic transmission by DA signaling (Snyder et al., 1998, 2005).

There are multiple modulatory effects following D2R activation. First of all, these receptors are coupled with Gi/o proteins and their activation negatively modulates cAMP signaling, reducing the phosphorylation of the downstream proteins (PKA targets), such as DARPP-32. At the same time, activation of D2R, via the Gβγ subunits, inhibits L-type Ca^2+^ channels and activates G-protein-coupled Inwardly Rectifying potassium (K^+^) channels (GIRK) causing a decrease of neuronal excitability and a reduction in the synthesis and release of DA (Kebabian and Greengard, 1971). Moreover D2Rs are also located presynatpically onto the excitatory inputs where influence glutamate release and on ChaT interneurons in the striatum where contribute to reducing Ach release (Surmeier et al., 2007).

Interestingly, DA has a lower affinity for D1Rs compared to D2Rs, pointing at a different effect on the direct and indirect pathway during tonic or phasic DA release. Indeed, it has been suggested that phasic release activates D1Rs to facilitate limbic inputs while tonic release bidirectional activates D2Rs on PFC inputs (Floresco et al., 2003; Goto and Grace, 2005; Goto et al., 2007). It is important to consider the different effects of DA change the functions of the brain regions that receive DA inputs. Indeed, an altered DA modulation of the excitatory inputs onto these regions plays an important role in the pathophysiology of many neurological disorders (Goto et al., 2007).

## DA modulation of NMDARs and AMPARs

DA modulates the functioning of the glutamatergic synapse by acting at different levels. The classical view indicates that DA can regulate the activity of ionotropic glutamate receptors with a reduction of AMPAR-evoked responses and an increase of NMDAR-evoked responses (Cepeda et al., 1993; Levine et al., 1996; Cepeda and Levine, 1998; Graham et al., 2009). In particular, activation of D1R usually leads to potentiation of NMDAR-dependent currents, while activation of D2R induces a decrease of AMPAR-dependent responses. This view has a key relevance in the striatum where dopaminergic terminals form synaptic contacts at the neck of MSN spines, while the head receives inputs from glutamatergic terminals (Surmeier et al., 2007).

Interestingly, NMDARs in the corticostriatal synapse show peculiar features. Indeed, even if GluN2B represents the predominant regulatory subunit expressed in this brain area (Dunah and Standaert, 2001), it has been proposed that GluN2A- but not GluN2B-containing NMDARs induce a depression of synaptic transmission that does not involve activation of corticostriatal neurons but it is rather mediated NMDARs at MSN synapses (Schotanus and Chergui, 2008a). Interestingly, recent reports have suggested that GluN2A and GluN2B subunits differentially contribute to the glutamatergic transmission in striatal MSNs (Paoletti et al., 2008; Jocoy et al., 2011). While genetic deletion or pharmacological blockade of GluN2A increase D1R-mediated potentiation of NMDAR-dependent responses, inhibition of GluN2B reduces this potentiation, suggesting a counterbalance of their respective functions. Moreover, it has shown that GluN2A subunits contribute mainly to NMDA responses in D1-MSNs, whereas GluN2B subunits is more involved in NMDA responses in D2R cells (Paoletti et al., 2008; Jocoy et al., 2011).

Several studies have investigated the effect of D1R stimulation on NMDAR subunit trafficking at the synaptic membrane. Pharmacological activation of D1R enhances NMDARs surface levels (Hallett et al., 2006; Paoletti et al., 2008) and NMDAR localization in the synaptosomal membrane fraction through stimulation of the tyrosine kinase Fyn (Dunah et al., 2004; Tang et al., 2007). In more detail, it has been shown that treatment with D1R agonist (SKF38393) leads to a significant decrease of GluN2A-containing NMDARs and to a concomitant increase in spine head width (Vastagh et al., 2012). Interestingly, co-treatment of corticostriatal slices with GluN2A antagonist (NVP-AAM077) and D1R agonist augmented the increase of dendritic spine head width observed with SKF38393 alone. Conversely, GluN2B antagonist (ifenprodil) blocked any morphological effect induced by D1 activation (Vastagh et al., 2012). However, further studies are still needed for a comprehensive understanding of the specific role of GluN2A- *vs*. GluN2B-containing NMDARs in the modulation of dendritic spine morphology at striatal MSNs.

BAC transgenic mice expressing EGFP in D1R- and D2R-positive cells (Valjent et al., 2009) has recently been used to carefully analyze DA-dependent modulation of MSNs within the direct and indirect pathways (Cepeda et al., 2008). In agreement with previous studies, D1R-dependent modulation of glutamate-evoked responses was correlated with the activation of direct pathway neurons. On the contrary, D2R-dependent reduction of glutamate-evoked responses was specific to the indirect pathway (André et al., 2010). Moreover, recent and advanced tools such as optogenetics and sophisticated Ca^2+^ imaging have shown that activation of D2 receptors decrease NMDAR-induced responses by presynaptic modulation of glutamate release (Higley and Sabatini, 2010).

Notably, several studies describing the co-existence of D1Rs and NMDARs at striatal MSN synapses indicate the presence of a possible direct molecular interaction between the two receptor systems (Kung et al., 2007; Heng et al., 2009; Kruusmägi et al., 2009; Jocoy et al., 2011; Vastagh et al., 2012). A direct interaction between these two receptors was originally proposed by Lee et al. (2002), who showed co-immunoprecipitation of D1R with GluN1/GluN2A subunits of the NMDAR. This interaction is not static, but is decreased by D1R activation (Lee et al., 2002; Luscher and Bellone, 2008). In addition, disruption of D1R interaction with GluN2A-containing NMDARs by interfering peptides is sufficient to induce a modulation of NMDAR currents thus suggesting a direct role for this receptor-receptor binding in NMDA-transmission (Lee et al., 2002; Brown et al., 2010). However, the issue is more complicated since in both striatal neurons and transfected HEK293 cells, D1R directly interacts with GluN1 subunit to form a constitutive oligomeric complex that is recruited to the plasma membrane by the presence of GluN2B subunit (Fiorentini et al., 2003). Moreover, this interaction abolishes D1R internalization, a crucial adaptive response that normally occurs upon agonist stimulation (Fiorentini et al., 2003).

More recent studies have applied high-resolution single nanoparticle live-imaging techniques to investigate the role of the dynamic interaction between D1R and NMDAR at hippocampal synapses (Ladepeche et al., 2013a). The prevention of the physical interaction between D1R and GluN1 by interfering peptide is able to fully abolished the synaptic stabilization of D1R, thus suggesting that D1Rs are dynamically retained at glutamatergic synapses through a mechanism requiring the interaction with NMDAR (Ladepeche et al., 2013a). Moreover, disruption of D1R/NMDAR complex increases NMDAR synaptic content through a fast lateral redistribution of the receptors, and favors long-term synaptic potentiation (Ladepeche et al., 2013b). In particular, D1R activation reduces D1R/GluN1 interaction at perisynaptic sites and allows the lateral diffusion of NMDARs into the postsynaptic density where they support the induction of Long-term potentiation (LTP; Argilli et al., 2008; Ladepeche et al., 2013b).

D2-type DA receptors also interact with NMDARs. At the postsynaptic density, D2Rs form a specific complex with the NMDARs through the C-terminal domain of GluN2B subunit (Liu et al., 2006). Interestingly, DA stimulation by cocaine (i) enhances the D2R/GluN2B interaction; (ii) reduces the association of CaMKII with GluN2B; (iii) lowers the CaMKII-dependent phosphorylation of GluN2B (Ser1303); and (iv) inhibits NMDA receptor-mediated currents in MSNs (Liu et al., 2006).

DA can also modulate the activity of AMPARs leading to a reduction of AMPAR-evoked responses (Cepeda et al., 1993; Levine et al., 1996; Cepeda and Levine, 1998; Bellone and Lüscher, 2006; Engblom et al., 2008; Mameli et al., 2009; Brown et al., 2010). Early studies performed in cultured neurons showed that activation of D1R in striatal MSNs promotes the phosphorylation of AMPARs by PKA as well as the potentiation of current amplitude (Price et al., 1999). D2Rs antagonists increase the phosphorylation of GluR1 at Ser845 without affecting the phosphorylation at Ser831 (Håkansson et al., 2006). The same effect is observed using eticlopride, a selective D2R antagonist. On the contrary, D2R agonist quinpirole decreased GluR1 phosphorylation at Ser845 (Håkansson et al., 2006). Modulation of DA receptors is also able to regulate AMPAR trafficking at the synaptic membranes. In particular, treatment with D1R agonist leads to an increase of AMPA receptor subunits surface expression (Snyder et al., 2000; Gao et al., 2006; Vastagh et al., 2012).

## DA modulation of synaptic plasticity

DA plays an important role in modulating long-term changes in synaptic strength. One of the best-characterized forms of synaptic plasticity in the striatum is the long-term depression (LTD). In the dorsal and ventral striatum this form of plasticity requires the concomitant activation of mGluR5 and voltage-gated calcium channels and it is expressed by the release of endocannabinoids (eCBs). eCBs act retrogradly onto their CB receptors and decrease the probability of glutamate release (Robbe et al., 2002; Kreitzer and Malenka, 2005).

Interestingly, this form of LTD depends upon the activation of D2Rs, but whether it is controversial whether is only expressed at glutamatergic inputs onto MSNs of the indirect pathway of the dorsal striatum. Indeed, while eCB-LTD has been first characterized in D2R MSNs of the dorsal striatum (Kreitzer and Malenka, 2007), this form of plasticity has been described in both D1R and DR2 striatal neurons of the direct and indirect pathways in BAC transgenic mice (Wang et al., 2006). One possible explanation for the expression of this form of LTD at MNS synapses that do not express D2Rs is that, in both cell types, D2R-dependence of LTD induction is not direct, but it rather depends upon the activation of D2Rs in cholinergic interneurons (Wang et al., 2006).

Long-term potentiation (LTP) at excitatory inputs onto MSNs in the dorsal and ventral striatum is less characterized, and the information that are available so far is even more controversial compared to striatal LTD because of the variety of protocols used to induce this form of plasticity by different laboratories. In the dorsal striatum, LTP induction onto D1 MSNs depends on D1Rs, while, in D2 MSNs, the same form of synaptic plasticity requires the activation of adenosine A2R (Shen et al., 2008; Pascoli et al., 2014a). In both the direct and indirect pathways, the activation of D1Rs and A2Rs, and the concomitant activation of NMDARs leads to the phosphorylation of DARPP-32 and MAPKs that are involved in the expression of LTP (Calabresi et al., 1992, 2000; Kerr and Wickens, 2001; Surmeier et al., 2014). In the ventral striatum, a protocol of High Frequency Stimulation (HFS) induces a form of LTP that relies on the activation of D1Rs but not D2Rs (Schotanus and Chergui, 2008b). Interestingly, previous work showed that LTP is impaired by both D1 and D2 antagonists suggesting that this form of LTP depends upon DA concentration (Li and Kauer, 2004). A recent study, using cell identification, reported that while HFS-LTP is induced in both D1 and D2 MSNs, this form of LTP is blocked by cocaine treatment only in the direct pathway (Pascoli et al., 2011b). The authors characterized the induction and expression mechanisms of this LTP which was reported to be NMDA and ERK pathway-dependent. Future studies are required to investigate the mechanisms underlying LTP in the indirect pathway, and to characterize this form of synaptic plasticity in an input specific manner.

The role of DA in governing striatal plasticity has been addressed by analyzing the mechanisms of Spike Time Dependent Plasticity (STDP) in the dorsal striatum. In both D1 and D2 MSNs, synaptic plasticity follows Hebbian rules. LTP is indeed induced when postsynaptic spiking follows synaptic activity (positive timing), while LTD is favored when the order is reversed (negative timing). Compared to other synapses, in the dorsal striatum, DA plays important roles in determining the sign of synaptic plasticity. In the direct pathway, positive timing gives rise to LTP only when D1 are stimulated, otherwise it leads to LTD. Instead, negative timing induces LTD when D1Rs are not stimulated. In the indirect pathway, D2 signal is necessary for LTD when the postsynaptic spiking is followed by synaptic stimulation. When D2Rs are blocked and A2Rs are stimulated, the same pairing protocol induces LTP (Shen et al., 2008). Therefore, DA modulation in the dorsal striatum ensures that the bidirectional synaptic plasticity follows the Hebbian rules. Further investigation is needed to determine whether these rules apply to all glutamatergic inputs and to the ventral striatum too.

## Parkinson disease

Parkinson’s disease (PD) physiopathology is linked to a widespread degeneration of DA-releasing neurons of the Substantia Nigra pars compacta (SNpc), with the loss of DA reaching striatal projecting neurons (Obeso et al., 2010). The degeneration of the nigrostriatal dopaminergic pathway leads to significant morphological and functional changes in the striatal neuronal circuitry, including modifications of the corticostriatal glutamatergic synaptic architecture (Sgambato-Faure and Cenci, 2012; Mellone and Gardoni, 2013) and the consequent loss of striatal synaptic plasticity (Calabresi et al., 2014). A very elegant study demonstrated the asymmetry of the effect of DA denervation on the connectivity of striatonigral and striatopallidal MSNs (Day et al., 2006). In particular, DA depletion leads to a profound decrease in dendritic spines and glutamatergic synapses on striatopallidal MSNs but not on striatonigral MSNs (Day et al., 2006).

It was recently shown that distinct degrees of DA denervation differentially affect the induction and the maintenance of two distinct and opposite forms of corticostriatal synaptic plasticity (Paillé et al., 2010). An incomplete (approximately 75%) nigral denervation does not affect corticostriatal LTD in MSNs, which is however abolished by a complete lesion. This result indicates that a low although critical level of DA is required for this form of synaptic plasticity. Conversely, an incomplete DA denervation dramatically alters the maintenance of LTP in MSNs, demonstrating a critical role of this form of synaptic plasticity in the early motor parkinsonian symptoms (Paillé et al., 2010). In two different models of PD Shen et al. (2008) showed that in D2R-expressing MSNs, LTP was induced not only by the usual pairing protocol but also with a validated protocol known to induce LTD. Conversely, in D1R-expressing MSNs a protocol normally inducing LTP produces a robust form of LTD that was sensitive to CB1 receptor block (Shen et al., 2008). Imbalances between neural activity in the direct vs. the indirect pathway have been indicated as a major event underlying severe motor deficits observed in PD (Calabresi et al., 2014). In models of PD, eCB-mediated LTD is absent but is rescued by treatment with D2R receptor agonist or with inhibitors of eCB degradation (Kreitzer and Malenka, 2007), thus indicating eCB-mediated depression of indirect-pathway synapses as a critical player in the control of motor behavior in PD.

Alterations of NMDAR subunit composition at MSNs synapses have been reported to sustain this altered expression of plasticity (Sgambato-Faure and Cenci, 2012; Mellone and Gardoni, 2013). It is known that NMDARs are characterized by GluN2A and GluN2B regulatory subunits in MSNs, being GluN2B the most abundant (Dunah and Standaert, 2001). Notably, changes in synaptic NMDAR GluN2A/GluN2B subunit ratio in striatal MSNs correlate with the motor behavior abnormalities observed in a rat model of PD (Picconi et al., 2004; Gardoni et al., 2006; Mellone and Gardoni, 2013). In particular, levels of GluN2B were specifically reduced in synaptic fractions from fully-lesioned 6-OHDA rats when compared to sham-operated rats in the absence of GluN2A alterations in the same samples (Picconi et al., 2004; Gardoni et al., 2006; Paillé et al., 2010). In addition, in the 6-OHDA model of PD, rats with a partial lesion of the nigrostriatal pathway (about 75%) showed a dramatic increase in the GluN2A immunostaining at the synapse without any modifications of GluN2B (Paillé et al., 2010). Overall these data indicate an increased GluN2A/GluN2B ratio at MSNs synapses at different stages of DA denervation in experimental rat models of PD. Accordingly, a cell-permeable peptide that interfers with the interaction between GluN2A and the scaffolding protein PSD-95 is able to reduce the synaptic levels of GluN2A-containing NMDARs and to rescue the physiological NMDAR composition and synaptic plasticity in MSNs (Paillé et al., 2010). Moreover, stimulation of D1Rs by systemic administration of SKF38393 normalizes NMDAR subunit composition and improves motor behavior in a model of early PD establishing a critical link between a specific subgroup of DA receptors and NMDARs and motor performances (Paillé et al., 2010).

**Figure 2:**
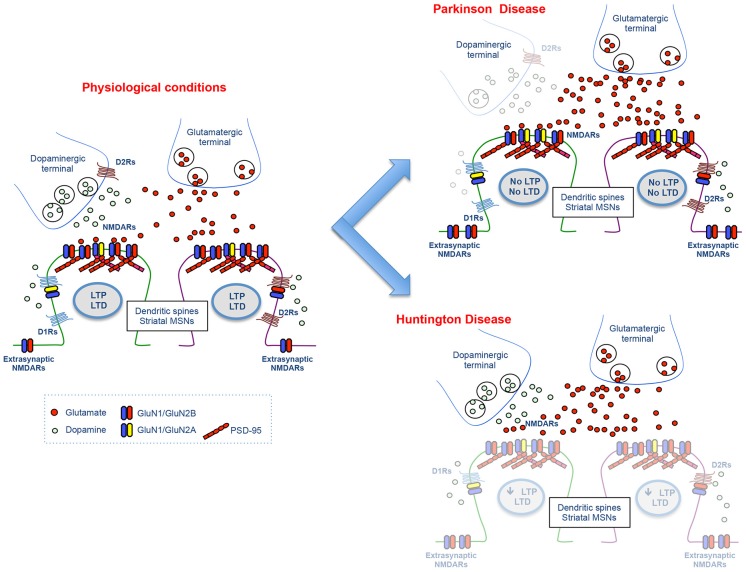
**Molecular and functional changes at the glutamatergic synapse in Parkinson and Huntington disease**. The cartoon illustrates the physiological glutamatergic corticostriatal synapse (left panel) and the molecular and functional alterations at DA and NMDA receptor level observed in experimental models of Parkinson and Huntington disease (right panels).

Altogether, the emerging pathophysiological picture shows that the strength of glutamatergic signals from the cortex to the striatum might be dynamically regulated by the different degree of DA denervation during the progression of the disease (Figure 2). In fact, bidirectional changes in corticostriatal synaptic plasticity are critically controlled by the degree of nigral denervation that influences the endogenous DA levels and the assembly of striatal NMDARs (Sgambato-Faure and Cenci, 2012).

## Huntington disease

Huntington’s disease (HD) is a progressive neurodegenerative disease which is characterized by chorea, cognitive decline, and psychiatric disturbances. Alterations in DA and DA receptor levels in the brain contribute to the clinical symptoms of HD (Spokes, 1980; Richfield et al., 1991; Garrett and Soares-da-Silva, 1992; van Oostrom et al., 2009). In particular, time-dependent modifications of DA signaling are correlated to biphasic alterations of the activity of the glutamatergic synapse (Cepeda et al., 2003; Joshi et al., 2009; André et al., 2011a). In agreement with this biphasic activity, Graham et al. (2009) demonstrated that susceptibility to NMDAR-dependent excitotoxicity in HD mouse models was correlated to the severity of their symptomatic stage. On the one hand, HD mice at an early age display enhanced sensitivity to excitotoxic NMDAR-dependent events compared to wild-type animals. On the other hand, old symptomatic HD mice are more resistant to NMDA-dependent neurotoxicity (Graham et al., 2009).

Dysfunction and loss of striatal MSNs represent the major neuropathological feature of the disease (Martin and Gusella, 1986). Although the mechanisms explaining a selective degeneration of MSNs in HD have not been addressed, several reports correlated an abnormal functioning of both dopaminergic and glutamatergic transmission to the induction of striatal MSNs death (Charvin et al., 2005; Fan and Raymond, 2007; Tang et al., 2007).

A decrease of D1R and D2R in striatum from postmortem HD brains has been reported in several studies (Joyce et al., 1988; Richfield et al., 1991; Turjanski et al., 1995; Suzuki et al., 2001). In addition, a significant alteration of both D1R and D2R density and function in the striatum has been described in HD mouse models (Bibb et al., 2000; Ariano et al., 2002; Paoletti et al., 2008; André et al., 2011b). Studies performed in knock-in HD striatal cells showed that mutant huntingtin enhances striatal cell death through activation of D1R but not D2R (Paoletti et al., 2008). Particularly, pretreatment with NMDA increased D1R-induced cell death of mutant but not wild-type cells thus suggesting that NMDARs potentiate the vulnerability of HD striatal cells to DA toxicity (Paoletti et al., 2008). Interestingly, an aberrant Cdk5 activity is involved in the augmented sensitivity of HD striatal cells to DA and glutamate inputs (Paoletti et al., 2008). In agreement with these data, Tang et al. (2007) reported that glutamate and DA act synergistically to induce elevated Ca^2+^ signals and to induce apoptosis of MSNs in HD mice. Again, these effects are selectively mediated by D1R and not by D2Rs (Tang et al., 2007). However, a role for D2R in mediating MSN degeneration has been put forward (Charvin et al., 2005, 2008), thus raising the hypothesis that both activation of D1R and D2R might contribute to glutamate/DA dependent toxicity. More recently, André et al. (2011b) showed that, at the early stage, glutamate release was increased onto D1R cells while it was unaltered onto D2R cells in HD mice. Notably, at the late stage, glutamate transmission was decreased onto D1R cells only. Overall, this study suggests that more changes occur in D1R cells than in D2R cells, at both presymptomatic and symptomatic ages. Finally, in agreement with this study, Benn et al. (2007) showed that the percentage of D2R-positive cells are not modified with the phenotype or with age. However, it must be taken into account that these results represent a clear discrepancy with early studies indicating a higher vulnerability of D2R in HD (Reiner et al., 1988; Albin et al., 1992). Accordingly, further studies are needed for a complete characterization and understanding of D1R vs. D2R alterations in HD.

Changes in synaptic vs. extra-synaptic localization of NMDARs are also crucial for neuronal survival in HD (Levine et al., 2010). In particular, a selective increase of striatal GluN2B-containing NMDARs in association with an early increase in extrasynaptic NMDAR signaling has been described in different HD animal models (Zeron et al., 2004; Milnerwood et al., 2010). In addition, excitotoxicity mediated by GluN2B-containing NMDARs exacerbated selective MSNs degeneration in a knockin HD model (Heng et al., 2009).

DA and glutamate cross-talk seems to have a key role also in aberrant synaptic plasticity which is observed in HD animal models. DA-dependent LTP, but not LTD, in the dorsal striatum is reduced in the R6/2 mouse model of HD (Kung et al., 2007; Figure 2). Interestingly, the deficits in LTP and short-term plasticity observed in animal models of HD are reversed by treatment with the D1R agonist SKF38393 (Dallérac et al., 2011).

## Addiction

Drug-evoked synaptic plasticity of glutamatergic synapses in the mesocorticolimbic system has been largely implicated in addictive behaviors (Luscher and Bellone, 2008) and DA neurons of the VTA are the point of convergence at which addictive drugs can alter the brain circuits (Brown et al., 2010). Drug-evoked synaptic plasticity has been characterized at excitatory input onto DA neurons of the VTA 24 h after a single injection of addictive drugs (Ungless et al., 2001; Bellone and Lüscher, 2006; Mameli et al., 2007; Yuan et al., 2013). Interestingly, it is induced by activation of D1/D5Rs and NMDARs (Ungless et al., 2001; Argilli et al., 2008) and it is expressed by insertion of GluN3A-containing NMDARs (Yuan et al., 2013) and GluA2-lacking AMPARs (Bellone and Lüscher, 2006). Moreover, it has been shown that the redistribution of glutamatergic receptors induced by cocaine in the VTA depends upon the action of cocaine on DA transporter (DAT) and that DA neurons activity itself is sufficient to induce drug-evoked synaptic plasticity at glutamatergic synapses (Brown et al., 2010). D1 signaling in the VTA is necessary for these adaptations suggesting that the convergence of DAergic/glutamatergic signaling in the VTA modifies the circuit at the synaptic level.

Interestingly, redistribution of glutamatergic transmission in the VTA is permissive for the expression of drug-evoked plasticity in the NAc and subsequent addictive behaviors. Indeed, deletion of GluN1 selectively in the DA neurons of the VTA abolishes both cocaine-evoked plasticity in the NAc (Engblom et al., 2008) and prevent reinstatement of self-administration (Mameli et al., 2009).

**Figure 3:**
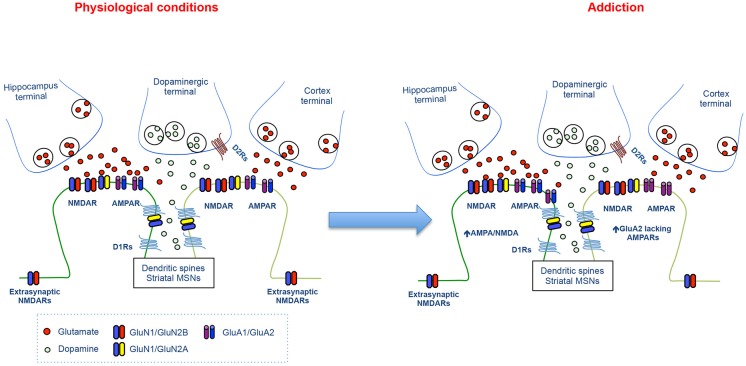
**Synaptic changes at the glutamatergic synapses during cocaine seeking**. The cartoon illustrates the physiological glutamatergic corticostriatal and hippocampastriatal synapses (left panel) and the synaptic alterations at excitatory synapses onto MSNs during cocaine seeking (Right panel).

In the NAc, the convergence of DA and glutamate after cocaine exposure contributes to addictive behaviors by the facilitation of AMPAR trafficking at certain glutamatergic inputs. Early studies have found that D1R stimulation increases GluA1 surface expression via PKA activation promoting further NMDA-dependent synaptic plasticity (Sun et al., 2005, 2008; Gao et al., 2006). Recently, the role of AMPAR trafficking in drug-evoked synaptic plasticity and its link to behavioral adaptation has been demonstrated. Indeed, insertion of GluA2-lacking (GluA1 homomeric) AMPARs has been shown both after incubation of cocaine craving and cocaine self-administration at excitatory input onto MSNs in the NAc (Conrad et al., 2008; Lee et al., 2013; Ma et al., 2014; Pascoli et al., 2014b; Figure 3). Although these studies show some discrepancies regarding the cell- and input-specificity of Ca^2+^ permeable AMPAR insertion, the removal of these receptors is an efficient method to revert addictive behaviors (Loweth et al., 2014; Pascoli et al., 2014b). Altogether, these studies indicate that the expression of addictive behaviors depends upon the convergence of DA/glutamate signal and the consequent changes in the efficacy and quality of excitatory synaptic transmission.

Which are the mechanisms underlying the interactions between the glutamate and the DA system in the NAc in drug addiction? Many studies have shown that different behavioral and molecular responses induced by cocaine rely on the D1R-NMDAR interaction that regulates the activity of ERK pathways and control gene expression, plasticity and behavior (Girault et al., 2007; Bertran-Gonzalez et al., 2008; Pascoli et al., 2014a). Interestingly, cocaine-induced activation of the ERK pathway is restricted to D1 MSNs and depends upon the concomitant activation of D1 and NMDARs. Moreover, direct blockade of ERK signaling induced by cocaine prevents the expression of conditioned place preference (CPP; Valjent et al., 2000), locomotor sensitization (Valjent et al., 2006) and drug-evoked synaptic plasticity (Pascoli et al., 2011b; Cahill et al., 2014). To confirm the role of DA/glutamate interaction in cocaine-induced ERK activation, it has also been shown that indirect inhibition of the ERK pathway blocks addictive behaviors. Cocaine activates the tyrosine kinase Fyn that, via phosphorylation of GluN2B, potentiates Ca^2+^ influx through NMDARs and activates ERK signaling. Interestingly, the inhibition of Fyn inhibits cocaine-induced ERK activation while inhibition of GluN2B-containing NMDAR impairs locomotor sensitization and CPP (Pascoli et al., 2011a). Moreover, the blockade of the D1/GluN1 downstream pathways, although it preserves the individual signaling, blocks both the D1-induced potentiation of Ca^2^+ influx via NMDARs and the ERK activation. As a consequence, behavioral sensitization is impaired (Cahill et al., 2014).

## Conclusions

Functional interactions between DA and glutamate receptors modulate an incredible variety of functions in the brain and, when abnormal, they contribute to numerous central nervous system disorders. In particular, an integrated cross-talk between DA and glutamate receptors plays a key role in motor control, cognition and memory, neurodegenerative disorders, schizophrenia and addictive behaviors. Accordingly, a huge number of studies, described in the present review, have been performed aiming at understanding the molecular and functional mechanisms coordinating functions of glutamate and DA receptors. Hopefully, a complete knowledge of dysregulation of glutamate and DA signaling as in Parkinson, Huntington and addiction-related diseases, could represent the first step for the identification and setting up of novel therapeutical approaches for these brain disorders.

## Conflict of interest statement

The authors declare that the research was conducted in the absence of any commercial or financial relationships that could be construed as a potential conflict of interest.
